# Risk and outcomes of adrenal crisis in primary adrenal insufficiency: Evidence from a 24‐year nationwide cohort study

**DOI:** 10.1111/joim.70153

**Published:** 2026-08-19

**Authors:** Ann‐Elin Meling Stokland, Lars Breivik, Ingvild Dalen, Sandra Dis Steintorsdottir, Simon Kildal, Ingrid Nermoen, Kari Lima, Margrethe Svendsen, Åse Bjorvatn Sævik, Aleksandra Debowska, Stina Therese Sollid, Daniel Hannisdal, Trine Elisabeth Finnes, Agnes Judit Horvath, Elin Korsgaard, Hallvard Singsås, Bjørn O. Åsvold, Maria Eriksson Steigen, Sigrid Blika, Petya Milova, Anders Svare, Anja Fog Heen, Lene Katrine Bjerke, Marianne Stedje, Siri Carlsen, Bjørn Gunnar Nedrebø, Anders Palmstrøm Jørgensen, Grethe Å. Ueland, Marianne Øksnes, Eystein Sverre Husebye

**Affiliations:** ^1^ Department of Endocrinology Stavanger University Hospital Stavanger Norway; ^2^ Department of Clinical Science University of Bergen Bergen Norway; ^3^ Department of Medicine Haukeland University Hospital Bergen Norway; ^4^ Department of Research Stavanger University Hospital Stavanger Norway; ^5^ Department of Endocrinology, Morbid Obesity and Preventive Medicine Oslo University Hospital Oslo Norway; ^6^ Division of Internal Medicine University Hospital of North Norway Tromsø Norway; ^7^ UiT The Arctic University of Norway Tromsø Norway; ^8^ Department of Endocrinology Akershus University Hospital, Nordbyhagen Lørenskog Norway; ^9^ Institute of Clinical Medicine Faculty of Medicine University of Oslo Oslo Norway; ^10^ Department of Medicine Østfold Hospital, Grålum Sarpsborg Norway; ^11^ Department of Internal Medicine Sørlandet Hospital Trust Arendal Norway; ^12^ Faculty of Health and Sport Sciences University of Agder Grimstad Norway; ^13^ Department of Medicine Vestfold Hospital Tønsberg Norway; ^14^ Department of Medicine Drammen Hospital, Vestre Viken Health Trust Drammen Norway; ^15^ Department of Medicine Bærum sykehus Gjettum Norway; ^16^ Clinic of Internal Medicine Innlandet Hospital Trust Hamar Norway; ^17^ Division of Medicine Vestre Viken Hospital Trust Kongsberg Norway; ^18^ Division of Medicine Levanger Hospital Levanger Norway; ^19^ Department of Endocrinology Clinic of Medicine St. Olavs Hospital Trondheim Norway; ^20^ HUNT Centre for Molecular and Clinical Epidemiology Department of Public Health and Nursing Norwegian University of Science and Technology Trondheim Norway; ^21^ Division of Medicine Telemark Hospital Notodden Norway; ^22^ Division of Medicine Telemark Hospital Skien Norway; ^23^ Division of Medicine Nord‐Trøndelag Hospital Trust Namsos Norway; ^24^ Department of Medicine Lovisenberg Diaconal Hospital Oslo Norway; ^25^ Department of Medicine Diakonhjemmet Hospital Oslo Norway; ^26^ Department of Medicine Fonna Hospital Trust Haugesund Norway

**Keywords:** adrenal crisis, autoimmune primary adrenal insufficiency, endocrine emergency, epidemiology, mortality, risk factors, type 1 diabetes

## Abstract

**Background:**

Adrenal crisis is a life‐threatening emergency. Despite preventive strategies, previous reports suggest increasing incidence and substantial mortality, but robust validated data are limited.

**Objective:**

To assess temporal trends in adrenal crisis incidence and identify associated risk factors.

**Methods:**

We studied 1040 patients with autoimmune or idiopathic primary adrenal insufficiency enrolled in the Norwegian Addison Registry. Medical records were reviewed for crisis‐related hospitalizations between 2000 and 2023. Overt adrenal crisis was defined by acute clinical deterioration with hemodynamic or biochemical abnormalities, whereas incipient crisis was defined by typical symptoms without objective abnormalities.

**Results:**

During a median follow‐up of 15 years, 660 patients (63%) experienced crisis‐related hospitalizations, and 265 (25%) had an overt adrenal crisis after diagnosis. The incidence of overt crises was 3.2 per 100 person‐years and that of incipient crises was 6.9 per 100 person‐years. Admission rates for incipient crises increased significantly over time (*p* < 0.001), whereas overt crisis rates remained stable. Among 1754 admissions, five deaths (0.3%) were attributed to adrenal crisis. Both younger and older age (*p* < 0.001) and type 1 diabetes (incidence rate ratio 2.09, 95% confidence interval 1.45–3.01; *p* < 0.001) were associated with increased overt crisis risk. Daily corticosteroid dose was not associated with crisis risk. Prehospital stress dosing was used in about 50% of admissions.

**Conclusions:**

Overt adrenal crisis was uncommon and crisis‐related in‐hospital mortality was exceptionally low. Crisis risk was independent of replacement dose but increased in patients with type 1 diabetes. Strengthening education and implementation of prehospital stress dosing may further reduce the burden of adrenal crises.

## Graphical abstract

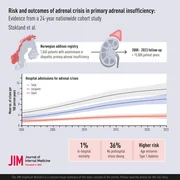

**Abbreviations**
AIC: Akaike information criterion
CA/HC: cortisone acetate/hydrocortisone
CAH: congenital adrenal hyperplasia
CI: confidence interval
DR‐HC: dual‐release hydrocortisone
IRR: incidence rate ratio
NorPD: Norwegian Prescribed Drug Registry
OR: odds ratio
PAI: primary adrenal insufficiency
RS: restricted cubic splines
ROAS: The Norwegian Addison Registry
T1D: type 1 diabetes

## Introduction

Primary adrenal insufficiency (PAI) is a chronic condition characterized by deficiency of glucocorticoids and mineralocorticoids, usually caused by autoimmune adrenal destruction [1]. Despite oral replacement therapy, patients remain at risk for adrenal crises, especially during intercurrent infections, trauma, and surgery. Adrenal crisis with hemodynamic instability is the most severe manifestation of adrenal insufficiency [2] and is considered a major contributor to premature mortality [3, 4, 5], reported in up to 6% of events [6]. However, no universally accepted definition of adrenal crisis exists, and diagnostic criteria vary across studies. Several reports suggest that hospital admissions related to adrenal insufficiency and adrenal crises have increased over the past two decades [7, 8, 9], in part associated with the wider use of short‐acting glucocorticoids [7, 9]. Although some risk factors for adrenal crisis have been described [2], large‐scale, detailed, and validated studies are lacking [10]. We therefore evaluated temporal trends in adrenal crisis incidence over 24 years, assessed in‐hospital mortality risk, and analyzed prespecified risk factors in a national patient registry.

## Methods

### Study design and population

We conducted a nationwide, registry‐based cohort study of patients with autoimmune or idiopathic PAI enrolled in the Norwegian Addison Registry (ROAS). Based on the most recent national coverage analysis, which incorporated data from the Norwegian Prescription Database (NorPD), ROAS captures 86% of patients with autoimmune or idiopathic PAI (2016–2025). Patients with PAI are generally followed lifelong by specialist endocrinology services through hospital‐based outpatient clinics.

A total of 1040 patients were included, after exclusion of 12 patients (4 who died before 2000, 1 moved abroad, and 7 with missing hospital records). Patient characteristics are summarized in Table 1. All diagnoses were validated against hospital records. Patients with PAI and antibodies against 21‐hydroxylase (*n* = 946) or with associated autoimmune diseases (*n* = 62) were classified as having autoimmune PAI. Idiopathic PAI was diagnosed in 32 patients (3%), including 26 with negative and 6 with unknown 21‐hydroxylase antibody status and no autoimmune comorbidities. The study was approved by the Ethics Committee of Western Norway (REK no. 551436). All participants provided informed consent at inclusion in ROAS (REK no. 14443).

**Table 1:** Clinical and epidemiologic characteristics of the 1040 included patients.

Basic demographics	
Women, *n* (%)	601 (58%)
Men, *n* (%)	439 (42%)
Age at diagnosis, median (range)	33 years (1–85)
Age at the end of follow‐up, median (range)	56 years (12–100)
Deceased during follow‐up, *n* (%)	156 (15%)
**Observation time, person‐years**	
Cumulative, total	15,221
Cumulative, excluding partially observed years	14,823
Age groups	
2–19 years	328
20–39 years	3610
40–59 years	6205
60–79 years	3926
80–99 years	754
Sex	
Female	8388
Male	6435
Type 1 diabetes	
Yes	2162
No	12,661
Medication type^a^	
CA/HC	6592
DR‐HC	1091
PAI registered in summary care record^b^	
Yes	2619
No	2691
**Autoantibodies, *n* (%)**	
21‐Hydroxylase antibodies	946 (94%)^c^
**Autoimmune comorbidity, *n* (%)** ^d^	
One or more comorbidities	769 (74%)
Type 1 diabetes	171 (16%)
Hypothyroidism	507 (49%)^e^
Graves’ disease	105 (10%)
Celiac disease	89 (9%)
Vitamin B12‐deficiency	121 (12%)
Vitiligo	95 (9%)
Hypoparathyroidism	33 (3%)
Primary ovarian insufficiency	71 (12%)^f^

Abbreviations: CA/HC, cortisone acetate/hydrocortisone; DR‐HC, dual‐release hydrocortisone.

^a^ Data before 2013 are excluded.

^b^ Data before 2017 are excluded.

^c^ Data are missing in 30 patients.

^d^ Numbers correspond to diagnoses made at any point during the course of disease.

^e^ Including autoimmune hypothyroidism and ablative treatment for Graves’ disease.

^f^ Women only.

### Data source, validation, and definitions

Hospital records of all patients were reviewed for admission due to adrenal crisis between January 1, 2000, and December 31, 2023. All data were collected by endocrinologists or attending physicians in endocrinology between January 2024 and April 2025. *Overt adrenal crisis* was defined as an acute deterioration in health status associated with at least one of the following criteria: prehospital or in‐hospital hypotension (systolic blood pressure <100 mmHg), hyponatremia (<130 mmol/L), hyperkalemia (>5 mmol/L) or hypoglycemia (<3.5 mmol/L). Isolated hyponatremia or hyperkalemia that could be attributed to other causes was not classified as overt crises. In patients with diabetes, glucose‐corrected sodium values were used, and hypoglycemia related to insulin administration was not considered a criterion for defining overt adrenal crisis alone. *Incipient adrenal crisis* was defined as typical symptoms including marked impairment of general health, nausea, vomiting, and abdominal pain in the absence of objective biochemical and hemodynamic features, with resolution of symptoms after parenteral glucocorticoid administration. In all cases, only hospital admissions requiring intravenous treatment with hydrocortisone were included. For each patient, the annual number of overt, incipient, and total (the sum of both) crises was recorded. For each crisis, clinical details at admission and the outcome (fatal or nonfatal) were documented. In cases of death, a detailed review of medical records was performed to determine whether the crisis was the primary cause. When a specific cause of death other than adrenal crisis was identified, it was recorded accordingly. If no alternative cause was found and the patient's condition before death was consistent with adrenal crisis, the death was classified as attributable to adrenal crisis. Age was defined for each year as the difference between calendar year and the patients’ year of birth. For patients with type 1 diabetes (T1D), the diagnosis year was recorded as the onset of diabetes. Sex (male or female) was registered at time of diagnosis.

### Glucocorticoid treatment and preventive measures

For all patients, the types of corticosteroids used during the observation period were recorded. For a representative subgroup of patients (*n* = 524) from three regions and 11 hospitals, detailed annual data on glucocorticoid type, dose, and fludrocortisone dose were extracted from hospital records for the years 2006–2023. All doses were converted to hydrocortisone equivalents (i.e., 25 mg cortisone equals 20 mg hydrocortisone). For patients who switched to dual‐release hydrocortisone (DR‐HC) (Plenadren) (*n* = 213), the year of switching was recorded. If the patient experienced an adrenal crisis during a given year, the glucocorticoid type and dose used at the time of the event were recorded. The Norwegian Summary Care Record is a national electronic system providing registration of critical patient information accessible to all Norwegian healthcare professionals. For each patient, we recorded whether PAI was documented as critical information, and if so, the date of registration. Prescription data on parenteral hydrocortisone were retrieved from the Norwegian NorPD, including information on the timing of retrieval of all medications between 2004 and 2023. Access to injectable hydrocortisone was defined as available from the date of retrieval and for the subsequent four or five years, reflecting the medication`s shelf‐life according to Norwegian Medical Products Agency. Further details are provided in Supporting Information Appendix p. 3.

### Statistical analysis

Descriptive statistics are presented as counts and percentages for categorical variables, and as medians and ranges for continuous variables. Annual incidence rates were assessed using mixed‐effects negative binomial models, including calendar year as a categorical predictor. Random intercepts accounted for correlation between repeated observations within patients. Model‐based mean numbers of crises per year were presented with pointwise 95% confidence intervals (CIs).

To explore potential time trends, the effect of calendar year was modeled nonlinearly using restricted cubic splines (RCS), using three knots that optimized the Akaike information criterion (AIC). Statistical significance of the time trend was evaluated using an overall Wald test of the spline terms. Additionally, we estimated the average annual change assuming a linear effect of calendar year on the log scale, which implies a constant relative change in the expected mean number of crises per year. Incidence rate ratios (IRRs) for a 1‐year increase were presented with 95% CIs and *p*‐values from Wald tests.

The effect of age on the expected number of crises was modeled nonlinearly using RCS with four knots (chosen to optimize AIC) and visualized in plots. For tabular presentation, we categorized age into approximately 20‐year intervals. Age effects were adjusted for year (modeled nonlinearly), and potential differences in age effects over time were assessed using interaction terms.

Associations with other potential risk factors (sex, T1D, medication type, medication dose, and use of summary care record) were adjusted for calendar year and age, both modeled nonlinearly.

Dichotomized outcomes (at least one crisis within a year–yes/no) were assessed using multilevel logistic regression. Model‐based estimated proportions were presented in plots, and odds ratios (ORs) comparing values of potential risk factors were presented with 95% CIs and Wald *p*‐values.

For each analysis, we included all available data and detailed the number of valid or missing cases. In the regression analyses, partially observed years (typically, year of diagnosis and year of death) were excluded. SPSS v 30, Stata v 18, and R v 4.3.3 within the RStudio environment (2024.12.1) were used for the statistical analyses.

## Results

A total of 1040 patients were included with a median follow‐up of 15 years (range 0.2–24 years) and a cumulative observation time of 15,221 person‐years (Table 1). Altogether 427 patients (41%) experienced one or more admissions with overt adrenal crises, and 453 patients (44%) had one or more admissions with incipient crises during the observation period. About one‐fifth of the patients (*n* = 220, 21%) experienced both types of crises, whereas 380 (37%) had no admissions. Excluding crises at disease onset, 265 (25%) patients had one or more overt crises, 447 patients (43%) had incipient crises, 173 (17%) experienced both, and 501 (48%) had neither overt nor incipient post‐diagnosis crises.

During the observation period, 691 overt and 1063 incipient crises were recorded, corresponding to incidence rates of 4.5 and 7.0 per 100 person‐years, respectively. Excluding debut crises, 487 overt and 1051 incipient crises were recorded, with incidence rates of 3.2 and 6.9 per 100 person‐years, respectively. On average, the total incidence rate of crises increased by 4% per year (IRR 1.04, 95% CI 1.03–1.05; *p* < 0.001) (Table 2). This pattern persisted after adjusting for age in the population (Supporting Information Appendix p. 15). The incidence of overt adrenal crises rose before 2014 but remained stable thereafter (Fig. 1 and Table 2), whereas the incidence rate of incipient adrenal crises increased throughout the observation period (*p* < 0.001). A transient decline was observed during the COVID‐19 pandemic.

**Table 2:** Associations between demographic and clinical variables and adrenal crisis risk.

	Number of crises			At least one crisis		
	n/n_obs	IRR (95% CI)	*p*‐value	n/n_obs	OR (95% CI)	*p*‐value
**Year**						
Any		1.04 (1.03, 1.05)	<0.001		1.04 (1.03, 1.05)	<0.001
Overt		1.03 (1.01, 1.05)	0.001		1.03 (1.01, 1.05)	0.001
Incipient		1.05 (1.04, 1.06)	<0.001		1.05 (1.04, 1.06)	<0.001
**Age group (year)** ^a^						
Any	1012/14,820		<0.001	1012/14,821		<0.001
2–19		2.46 (1.58, 3.82)	<0.001		2.23 (1.37, 3.64)	0.001
20–39		1.88 (1.56, 2.27)	<0.001		1.91 (1.56, 2.34)	<0.001
40–59		1 (ref)			1 (ref)	
60–79		1.13 (0.93, 1.37)	0.20		1.09 (0.89, 1.34)	0.40
80–99		2.22 (1.62, 3.06)	<0.001		2.26 (1.59, 3.20)	<0.001
Overt	1012/14,821		<0.001	1012/14,821		<0.001
2–19		2.51 (1.25, 5.04)	0.010		2.88 (1.42, 5.84)	0.003
20–39		2.03 (1.50, 2.74)	<0.001		2.08 (1.52, 2.85)	<0.001
40–59		1 (ref)			1 (ref)	
60–79		1.32 (0.95, 1.82)	0.094		1.26 (0.90, 1.77)	0.17
80–99		3.26 (2.02, 5.25)	<0.001		3.23 (1.96, 5.31)	<0.001
Incipient	1012/14,820		<0.001	1012/14,820		<0.001
2–19		2.39 (1.45, 3.95)	0.001		2.29 (1.34, 3.93)	0.003
20–39		1.77 (1.43, 2.20)	<0.001		1.84 (1.47, 2.31)	<0.001
40–59		1 (ref)			1 (ref)	
60–79		1.05 (0.85, 1.31)	0.64		1.02 (0.81, 1.29)	0.86
80–99		1.91 (1.32, 2.76)	0.001		2.12 (1.43, 3.13)	<0.001
**Sex (female)** ^b^						
Any	1012/14,820	1.27 (1.03, 1.57)	0.027	1012/14,821	1.24 (1.00, 1.55)	0.051
Overt^c^	1012/14,821	1.13 (0.82, 1.55)	0.45	1012/14,821	1.13 (0.82, 1.56)	0.45
Incipient^c^	1012/14,820	1.26 (1.00, 1.58)	0.050	1012/14,820	1.21 (0.95, 1.53)	0.12
**Diabetes mellitus type 1** ^b^						
Any	1012/14,820	1.51 (1.17, 1.94)	0.002	1012/14,821	1.49 (1.14, 1.95)	0.004
Overt	1012/14,821	2.09 (1.45, 3.01)	<0.001	1012/14,821	1.98 (1.35, 2.90)	<0.001
Incipient	1012/14,820	1.37 (1.04, 1.82)	0.028	1012/14,820	1.41 (1.05, 1.89)	0.024
**Dual release hydrocortisone vs. cortisone acetate/hydrocortisone** ^b^ ^,^ ^d^						
Any^c^	925/7682	1.29 (0.99, 1.68)	0.057	925/7683	1.35 (1.01, 1.81)	0.046
Overt^c^	925/7683	1.06 (0.67, 1.69)	0.80	925/7683	0.85 (0.50, 1.43)	0.53
Incipient	925/7682	1.41 (1.06, 1.88)	0.020	925/7682	1.53 (1.13, 2.08)	0.007
**Registered in summary care record** ^b^ ^,^ ^e^						
Any^c^	859/5240	1.13 (0.89, 1.44)	0.32	859/5240	1.04 (0.80, 1.36)	0.77
Overt	859/5240	0.57 (0.37, 0.88)	0.011	859/5240	0.60 (0.37, 0.96)	0.032
Incipient^c^	859/5240	1.42 (1.09, 1.86)	0.011	859/5240	1.35 (1.01, 1.80)	0.042

*Note*: Results were estimated using multilevel negative binomial and binary logistic regression analyses. The multi‐level models included patient specific random intercepts. All p‐values are from Wald tests. The numbers of included patients/observations are indicated for each analysis.

Abbreviations: CI, confidence interval; IRR, incidence rate ratio; *n*, number of patients; n_obs, number of observations; OR, odds ratio; y, years of age.

^a^ Adjusted for year (modeled nonlinearly using restricted cubic splines with three knots).

^b^ Adjusted for year (nonlinear) and age (nonlinear; four knots).

^c^ Statistically significant interactions with year (nonlinear), see details in Supporting Information File.

^d^ Patients using cortisone acetate/hydrocortisone and dual‐release hydrocortisone from 2013 onward were included.

^e^ The analysis includes observations from 2017 onward.

**Fig. 1:**
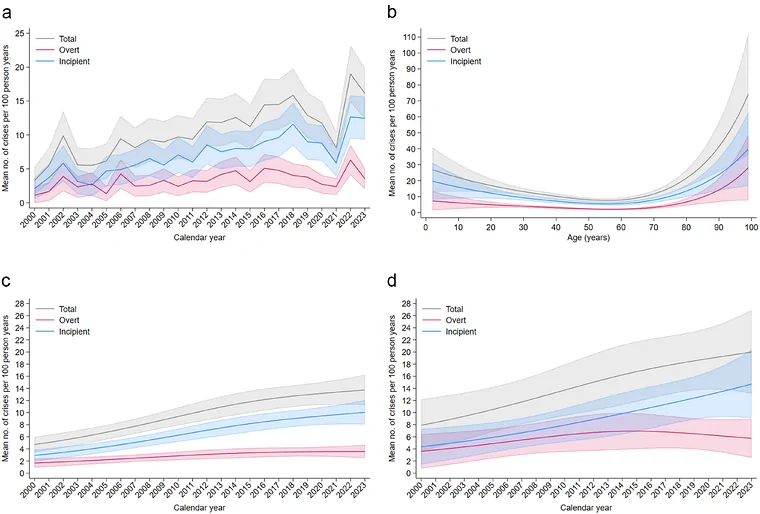
Rates of adrenal crises. Panel a shows observed incidence rates (per 100 person‐years) of overt adrenal crisis (red), incipient adrenal crisis (blue), and total crises (black) from 2000 to 2023. Panel b displays modeled incidence rates (per 100 person‐years) of overt adrenal crisis (red), incipient adrenal crisis (blue), and total crises (black) according to age from 2000 to 2023. Panels c and d show modeled incidence rates (per 100 person‐years) of overt adrenal crisis (red), incipient adrenal crisis (blue), and total (black) in patients without (c) and with (d) type 1 diabetes.

Out of 1754 registered admissions, 19 had a fatal outcome (1%; 15 involved overt crises and four involved incipient crises). Adrenal insufficiency was considered a contributing cause in five cases (0.3%), including three with cardiac arrest either in the immediate prehospital setting or upon arrival in the emergency room. There were no crisis‐related deaths registered after 2016. In the remaining cases, cardiovascular disease, chronic obstructive pulmonary disease, insulin‐induced hypoglycemia, and infection were considered the primary causes of death. Altogether, 156 patients (15%) died during the observation period. Median age at death was 77 years (range 19–100 years).

Infection was the most common precipitating cause (Fig. 2a), with gastroenteritis being the most frequent type of infection, followed by lower respiratory tract infections (Fig. 2b). In patients with known PAI the majority had normal electrolyte levels at admission; severe hyponatremia and hyperkalemia were uncommon (Table 3, Supporting Information Appendix p. 4). Overall, children younger than 16 years of age had a higher frequency of hyponatremia (34%) and hypoglycemia (15%). The proportion of admissions meeting the various diagnostic criteria for overt crisis is shown in the Supporting Information.

**Fig. 2:**
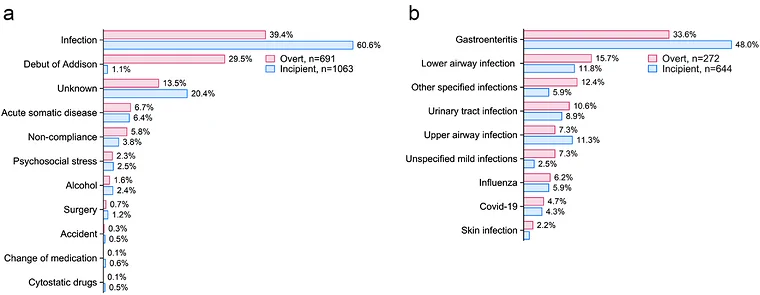
Precipitating causes of adrenal crisis: The data represent admissions for adrenal crises (n = 1754) with overt crises shown in red and incipient crises shown in blue. Panel a depicts the main precipitating causes. Panel b details the types of precipitating infection, based on data from 916 admissions classified as infection‐related.

**Table 3:** Clinical characteristics at admission.

	All admissions	Debut of PAI	Known PAI
Admissions, *n*	1754	216	1538
**Systolic blood pressure**			
mmHg, median (range)^a^	116 (40–219)	95 (50–171)	119 (40–219)
Hypotension, *n* (%)^b^	425 (24%)	123 (57%)	302 (20%)
Missing data, *n* (%)	48 (3%)	12 (6%)	36 (2%)
**S‐sodium**			
mmol/L, median (range)^a^	137 (98–154)	125 (100–141)	137 (98–154)
<130 mmol/L, *n* (%)	295 (17%)	157 (73%)	138 (9%)
<120 mmol/L, *n* (%)	84 (5%)	65 (30%)	19 (1%)
Missing data, *n* (%)	113 (6%)	7 (3%)	106 (7%)
**S‐potassium**			
mmol/L, median (range)^a^	4.0 (2.2–8.3)	5.0 (3.5–7.8)	4.0 (2.2–8.3)
>5 mmol/L, *n* (%)	224 (13%)	101 (47%)	123 (8%)
>7 mmol/L, *n* (%)	20 (1%)	5 (2%)	15 (1%)
Missing data, *n* (%)	136 (8%)	11 (6%)	125 (8%)
**S‐glucose**			
mmol/L, median (range)^a^	5.7 (0.6–57.2)	5.0 (1.4–30.9)	5.9 (0.6–57.2)
<3.5^c^ mmol/L, *n* (%)	74 (4%)	20 (9%)	54 (4%)
<2.0^c^ mmol/L, *n* (%)	28 (2%)	8 (4%)	20 (1%)
Missing data, *n* (%)	289 (16%)	42 (19%)	247 (16%)

*Note*: Data are presented as median (range) or *n* (%). Percentages include all admissions, as missing values were typically recorded as “normal” without a documented value.

^a^ Measured at admission.

^b^ Prehospital or in‐hospital.

^c^ Includes patients with diabetes.

The risk of adrenal crises was age‐dependent (*p* < 0.001) (Table 2). Adjusting for the effects of years of observation, the youngest age group (2–19 years) had on average 2.5 times more overt crises than the reference group (40–59 years) (IRR 2.51, CI 1.25–5.04; *p* = 0.010). Young adults (20–39 years) and those older than 80 years of age were also at increased risk of overt crises compared with the reference group, with estimated IRRs of 2.03 (95% CI 1.50–2.74; *p* < 0.001) and 3.26 (95% CI 2.02–5.25; *p* < 0.001), respectively. The admission rates were similar in women and men; however, women had a higher mean number of total admissions (IRR 1.27, 95% CI 1.03–1.57; *p* = 0.027) throughout the observation period (Table 2, Supporting Information Appendix p. 6).

Patients with T1D had a higher risk of overt adrenal crisis with an estimated IRR of 2.09 after adjusting for age (95% CI 1.45–3.01; *p* < 0.001) (Table 2). Among patients with T1D, 48 of 156 (31%) had one or more hospital admissions for severe hypoglycemia, whereas data were unavailable for 15 patients (9%). Twenty‐eight patients (18%) had two or more admissions with severe hypoglycemia, and 11 (7%) had five or more admissions. Thirty‐nine of 156 patients (25%) had one or more admissions with ketoacidosis. Over the observation period, median HbA1c levels decreased significantly comparable to patients with T1D in the Norwegian Diabetes Registry (Supporting Information Appendix p. 7).

During the observation period, 733 (71%) patients were treated exclusively with short‐acting hydrocortisone or cortisone acetate, whereas 213 (21%) received DR‐HC, and 43 (4%) received prednisolone or dexamethasone either alone or in combination with cortisone acetate. Forty‐one patients (4%) were treated with three or more different glucocorticoid preparations during their disease course. The proportion of DR‐HC users increased steadily over the observation period, and overall, they exhibited an increased risk of incipient crises (IRR 1.41, 95% CI 1.06–1.88; *p* = 0.02). However, this increased risk was confined to the period shortly after DR‐HC was introduced in Norway (2012), and the risk later diminished. Toward the end of the study, DR‐HC users had a lower risk of overt crises compared with those using short‐acting hydrocortisone or cortisone acetate (Fig. 3b, Supporting Information Appendix p.9). Corticosteroid and fludrocortisone dosages had no overall impact on the risk of adrenal crises (Table 2). Maintenance glucocorticoid dose and formulation were similar in patients with overt and incipient adrenal crises.

**Fig. 3:**
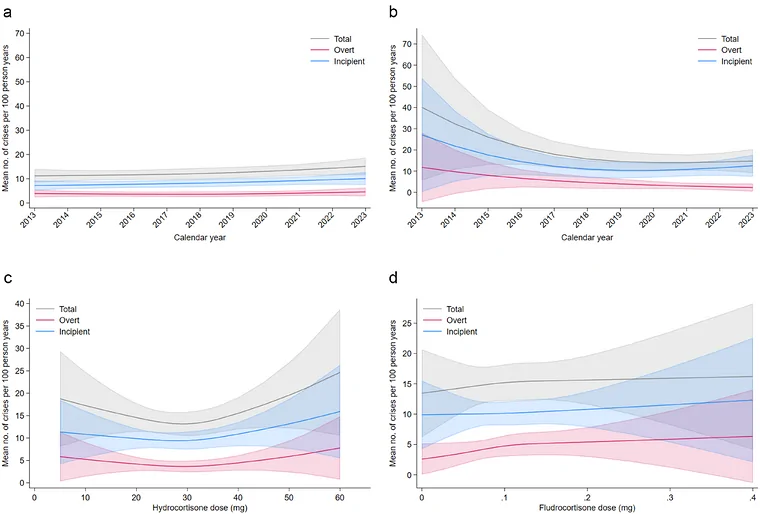
Adrenal crisis risk and treatment of primary adrenal insufficiency (PAI). Panel a shows incidence rates (per 100 person‐years) of overt adrenal crisis (red) and incipient adrenal crisis (blue) among patients treated with cortisone acetate/short‐acting hydrocortisone. Panel b shows the corresponding data for dual‐release hydrocortisone. Patients using a cortisol pump, prednisolone, or dexamethasone were excluded because of low numbers of observations. Panel c displays incidence rates (per 100 person‐years) of overt adrenal crisis (red), incipient adrenal crisis (blue), and total crises (black) according to glucocorticoid dose. Patients treated with cortisone acetate, hydrocortisone, and dual‐release hydrocortisone were included. Doses were converted to hydrocortisone equivalents in patients receiving cortisone acetate. Data were truncated at 60 mg. Children younger than 16 years of age were excluded. Panel d depicts incidence rates (per 100 person‐years) of overt adrenal crisis (red), incipient adrenal crisis (blue), and total crises (black) according to fludrocortisone dose. Data were truncated at 0.4 mg.

Although patients receiving hydrocortisone dosages below 15 mg tended to have an increased risk (Fig. 3c), very few patients were treated with such low doses (69 person‐years, children younger than 16 years were excluded from this analysis).

Among patients with available information, 88% had been issued a cortisol deficiency card during the course of their disease, and 81% had PAI registered in the National Summary Care Record. Having PAI registered as critical information in the Summary Care Record was associated with a reduced risk of overt adrenal crisis (IRR 0.57, 95% CI 0.37–0.88; *p* = 0.032) and with an increased likelihood of admissions for incipient adrenal crisis (IRR 1.42, 95% CI 1.09–1.86; *p* = 0.042) (Table 2, Supporting Information Appendix p.10).

A predictive model of hydrocortisone access showed that female sex, shorter disease duration, and younger age were associated with an increased likelihood of hydrocortisone retrieval (Supporting Information Appendix pp. 11–12). There was a large increase in patients with potential access to hydrocortisone during the observation period (2007–2023), with an absolute increase of 34 percentage points (95% CI 29–37; *p* < 0.001), OR 4.59 (95% CI 3.73–5.66; *p* < 0.001) (Supporting Information Appendix p. 12). Excluding crises at debut, 755 (49%) had received some form of prehospital treatment (oral and/or injectable hydrocortisone). In 553 admissions (36%), no stress dosing was administered, and in 230 admissions (15%), treatment status was unknown (Fig. 4). During the study period, the predicted probability of receiving any prehospital treatment increased by 21 percentage points between 2000 and 2023 (95% CI 12–31; *p* < 0.001), corresponding to an OR of 2.51 (95% CI: 1.58–3.80) (Supporting Information Appendix pp. 13–15).

**Fig. 4:**
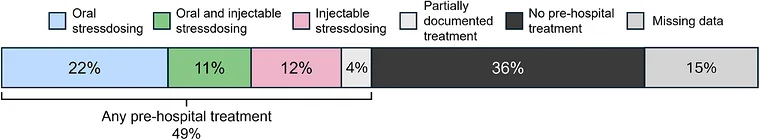
Prehospital stress dosing. Distribution of prehospital treatment for 1538 admissions, excluding crises at disease onset. Cases with at least one confirmed treatment were classified by known treatment type and are shown in blue (oral stress dosing), red (injectable hydrocortisone), green (both treatments), or light gray (one treatment type recorded, other unknown). Black displays cases with no prehospital treatment administered, and dark gray represents cases with completely unknown treatment status. The brackets indicate the total proportion of patients receiving any prehospital treatment.

## Discussion

In this large nationwide cohort study with 24 years of follow‐up, we found that the incidence of hospital‐treated overt adrenal crisis remained low and stable, whereas the incidence of incipient adrenal crisis was higher and increased over time. Crisis‐related in‐hospital mortality was nearly absent, underscoring early admission as a key determinant of favorable outcome. Notably, 75% of patients did not experience any episodes of overt adrenal crisis after diagnosis. The highest risk was observed among the youngest and oldest age groups and among individuals with T1D. The overall risk of adrenal crises was independent of corticosteroid dose and type, except during the initial years following the introduction of DR‐HC. Over the observation period, the proportion of patients collecting injectable hydrocortisone increased, but stress dosing before admission was only documented in half of the crisis episodes.

The incidence of overt adrenal crisis observed in our study was lower than previously reported in patients with PAI, where rates ranging from 5.2 to 24 per 100 person‐years have been reported [6, 11, 12, 13, 14, 15, 16]. Several factors may explain this discrepancy. Reliance on International Classification of Diseases coding, national databases [9, 12, 13] or patient‐reported outcomes [6, 17, 18, 19] may have reduced specificity and accuracy. Many studies were based on few patients from a single center, introducing potential selection bias. Our approach focusing on a well‐defined population included in a national registry and conducting a systematic review of hospital records for every admission supports the conclusion that the true incidence of overt adrenal crises is lower than previously thought. However, the results should be interpreted within the context of a universal healthcare system with broad access to specialist care and may not be directly applicable to patients with secondary adrenal insufficiency who generally experience lower crisis rates [15].

Another notable finding was the exceptionally low in‐hospital mortality associated with adrenal crisis (0.3%). This contrasts with the prospective 2‐year study by Hahner et al., which reported 64 episodes of adrenal crisis among 423 patients and 10 crisis‐related deaths [6]. One possible explanation is their use of interviews with relatives to ascertain the causes of death; another is the inclusion of patients with secondary adrenal insufficiency. Our data indicate that patients who reach hospital care almost invariably survive, and that crisis‐related mortality occurs predominantly outside the hospital or in patients with undiagnosed PAI. Our findings are also consistent with recent reports suggesting favorable long‐term survival among patients with autoimmune PAI [20].

When examining temporal trends, we observed a significant increase in incipient crises, whereas the incidence of overt crises remained relatively stable throughout the observation period. Rushworth et al. also reported a marked increase in hospital admissions for adrenal crisis between 2000 and 2019. However, they did not distinguish between primary and secondary adrenal insufficiency, and the diagnoses were not validated [7]. Similarly, Gafner et al. observed increasing adrenal crisis‐related hospitalizations in Switzerland between 2012 and 2022, with the steepest increase among patients with secondary adrenal insufficiency [21]. During the study period, cortisol deficiency cards and summary care records were introduced in Norway, and several centers implemented patient education programs. Notably, the inclusion of the PAI diagnosis in the summary care records was associated with a significant increase in admissions for incipient crises. This trend, alongside an overall heightened focus on preventive strategies, likely contributed to earlier hospital presentations, which may have mitigated progression to overt crises and reduced mortality among our patients.

Although we reaffirm advanced age as a risk factor for adrenal crisis [21, 22], our findings also reveal that the youngest patients are at increased risk. Other studies have not demonstrated an association with age; however, their findings are limited by small sample sizes and underrepresentation of younger and older age groups^11^, ^14^. Overall, epidemiologic data on adrenal crisis in younger populations remain scarce, and much of the existing literature concerns cases of non‐autoimmune PAI [23, 24]. Reisch et al. reported that 70% of adrenal crises in patients with congenital adrenal hyperplasia (CAH) occurred within the first 10 years of life, followed by a decline with age and a paradoxical increase among individuals aged 18–25 years [25], consistent with our findings. Diagnosis of adrenal crisis in children may be more challenging, as hypotension is more difficult to identify [2]. The lack of a specific definition of adrenal crisis in children may have led to underestimation of overt adrenal crises in our study. Among older patients, age‐related increases in blood pressure can obscure hypotension, whereas higher rates of infection and comorbidities may elevate risk^22^. Taken together, these findings suggest that current preventive strategies may not adequately address the needs of the youngest and oldest patients.

T1D was a common comorbidity in our population (16%) and was associated with an overall higher risk of overt adrenal crisis, consistent with the findings of Meyer et al. [13]. Balancing corticosteroid replacement while maintaining optimal glycemic control presents a challenge for both patients and clinicians. Our observation of relatively good glycemic control, together with a high incidence of severe hypoglycemia, and increased crisis risk, raises the question of whether these patients may benefit from individualized treatment targets and tailored glucocorticoid regimens.

Rising rates of adrenal crisis have previously been attributed to the current trend of lower replacement doses of short‐acting glucocorticoids [2, 7, 8, 9]. However, this assumption was based on epidemiologic data and glucocorticoid prescription rates, without accounting for the actual doses used [8, 9]. We found that lower normal doses of corticosteroid did not increase risk consistent with reports on patients with CAH [26]. Although users of DR‐HC initially had higher rates of incipient crises compared with those receiving conventional therapy, this difference diminished over time as clinical experience accumulated. Taken together, our findings support the use of relatively low replacement doses without increasing the risk of adrenal crises.

Remarkably, despite patient education efforts, only about 50% of admissions had documented use of any prehospital glucocorticoid stress dosing, aligning with previously reported figures [27]. In the prospective study by Burger‐Stritt et al., the median time from presenting the emergency card to a medical professional to the parenteral administration of hydrocortisone was 60 min, highlighting the critical importance of patients being able to self‐inject [28].

Although the proportion of patients obtaining injectable hydrocortisone from pharmacies has increased, a substantial proportion (especially men, older individuals, and those with long‐standing disease) still do not obtain it. The observation that relatively few patients receive appropriate stress dosing, combined with gastroenteritis remaining a leading precipitant of crisis, highlights the need to strengthen education on prehospital stress dosing for patients, family members, and ambulance personnel. Because infections are a frequent trigger, vaccination represents another important preventive measure.

The strengths of this study include a large, homogeneous, and well‐characterized cohort, recruited from all endocrine centers in Norway, making it representative for patients with autoimmune PAI in industrialized countries. Unlike prior studies, diagnostic accuracy was ensured through systematic case validation, and adrenal crises were classified using predefined objective criteria, and follow‐up was long and complete.

We acknowledge that the retrospective design is a limitation. For example, rates of prehospital treatment may have been underestimated if patients did not report administration upon admission. Indication bias may affect analyses of corticosteroid type and dose, as patients with a history of frequent adrenal crises may be more likely to receive higher replacement doses or be switched to DR‐HC. However, missing data were limited, and the risk of indication bias was mitigated by reporting actual drug use at each event. The lack of detailed data on hypoglycemic events and modern diabetes technologies limited our ability to explore determinants of adrenal crisis risk in patients with T1D. Finally, in‐hospital mortality in our study does not account for patients who died before reaching hospital care or at disease onset, as such cases would not have been recruited to the registry. Consequently, our estimates reflect hospital‐treated adrenal crises among registry‐enrolled patients.

In conclusion, admission rates for overt adrenal crisis remain low and stable, whereas rates for incipient crises have increased significantly. This trend likely reflects greater awareness among patients and clinicians, rather than the current use of low‐dose short‐acting glucocorticoids. Nevertheless, our findings highlight the need to improve patient education and management among the youngest and oldest patients and those with concomitant T1D. A critical measure to further reduce the incidence of overt crises is ensuring appropriate prehospital stress dosing.

## Author contributions

Ann‐Elin Meling Stokland, Lars Breivik, Grethe Å. Ueland, Marianne Øksnes and Eystein Sverre Husebye planned and designed the study. Ann‐Elin Meling Stokland and Lars Breivik coordinated the study. Ann‐Elin Meling Stokland, Ingvild Dalen, Lars Breivik and Eystein Sverre Husebye had direct access to and verified the original data. Ann‐Elin Meling Stokland, Ingvild Dalen and Lars Breivik performed the statistical analyses and generated the figures and tables. Ann‐Elin Meling Stokland wrote the first draft of the manuscript. Ann‐Elin Meling Stokland, Lars Breivik and Ingvild Dalen contributed equally to the final version of the work. Eystein Sverre Husebye edited the manuscript and supervised the conduct of the study. All other authors contributed to data collection, data analysis or interpretation, and revised the manuscript. All authors had final responsibility for the decision to submit the manuscript for publication.

## Funding information

This study was supported by the Health Authorities of Western Norway and The Research Council of Norway (grant no 335093).

## Conflict of interest statement

The authors declare no conflicts of interest.

## Ethics statement

The study was approved by the Regional Committee for Medical and Health Research Ethics (REK no. 551436). All participants gave informed consent upon inclusion in ROAS (REK no. 14443).

## Data sharing

We can share deidentified individual participant data underlying the results reported in this article, including the study protocol and informed consent form. Data will be available after publication of our manuscript upon request from investigators whose proposed use of the data has been approved by an independent review committee identified for this purpose. All requests should be sent to the corresponding author. The study group will evaluate all request and decide, based on the scientific rigor of the proposal, whether data sharing is appropriate.

## Supporting information




**Figure S1**. Serum sodium and potassium in adrenal crisis.
**Figure S2**. Diagnostic criteria of overt adrenal crisis.
**Figure S3**. Incidence of adrenal crisis by sex.
**Figure S4**. Glycemic control in type 1 diabetes over time.
**Figure S5**. Crisis risk by glucocorticoid type (cortisone/hydrocortisone vs. dual‐release hydrocortisone).
**Figure S6**. Crisis risk according to hydrocortisone dose.
**Figure S7**. Effect of Summary Care Record status on crisis risk.
**Figure S8A**. Predicted probability of access to injectable hydrocortisone.
**Figure S8B**. Probability of access to injectable hydrocortisone.
**Figure S8C**. Access to injectable hydrocortisone by age and sex.
**Figure S9**. Prehospital stress dosing.
**Table S1**. Summary of regression analyses.

## Data Availability

Data are not publicly available owing to national data protection regulations but may be made available from the corresponding author upon reasonable request.
