# Supplementary material for: Risk and outcomes of adrenal crisis in primary adrenal insufficiency: Evidence from a 24‐year nationwide cohort study
Source: J Intern Med. 2026 Aug 19;300(5):675–88. doi: 10.1111/joim.70153 (PMC13641282; doi:10.1111/joim.70153)
Supplement: Supplementary file 1 [file JOIM-300-675-s001.docx]

**Supplementary Appendix**

Supplement to: Meling Stokland AE, Breivik L, Dalen I, et al.

This appendix has been provided by the authors to give readers additional information about the work.

**Table of Contents**

Supplementary Methods

Figure S1. Serum sodium and potassium in adrenal crisis

Figure S2. Diagnostic criteria of overt adrenal crisis

Figure S3. Incidence of adrenal crisis by sex

Figure S4. Glycemic control in type 1 diabetes over time

Figure S5. Crisis risk by glucocorticoid type (cortisone/hydrocortisone vs. dual‑release hydrocortisone)

Figure S6. Crisis risk according to hydrocortisone dose

Figure S7. Effect of Summary Care Record status on crisis risk

Figure S8A. Predicted probability of access to injectable hydrocortisone

Figure S8B. Probability of access to injectable hydrocortisone

Figure S8C. Access to injectable hydrocortisone by age and sex

Figure S9. Prehospital stress dosing

Table S1. Summary of regression analyses

**Supplementary Methods, Further Details**

**Definition and Inclusion Criteria of Adrenal Crisis:**

Admissions with localized abdominal pain attributed to a known somatic cause but without additional symptoms or objective findings suggestive of an adrenal crisis were excluded. Admissions with symptoms of gastroenteritis were included because these symptoms cannot be reliably distinguished from those of an adrenal crisis. Admissions occurring abroad that could not be verified were excluded. When exact values of systolic blood pressure, serum sodium, serum potassium or serum glucose were missing but described as normal, the event was classified accordingly.

**Observation Period:**

When a patient changed home address during follow‑up, records were retrieved from the corresponding hospital. Each patient's observation period extended from January 1, 2000, or the date of diagnosis, until December 31, 2023, or death, whichever occurred first. If the exact date of diagnosis was unknown, July 1 was assigned; this applied to 11 patients diagnosed after 2000. Any periods in which the patient’s residence, follow-up location, or hospital records could not be determined or were incomplete were excluded.

**PAI Treatment:**

For all patients, the types of glucocorticoids used during the observation period were recorded. For a subgroup of patients (n=524) from three regions and elleven hospitals, the exact glucocorticoid type, dose and fludrocortisone dose were recorded annually from 2006 through 2023 based on hospital records. All doses were converted to hydrocortisone equivalents (i.e. 25 mg cortisone = 20 mg hydrocortisone). In addition, for all patients who switched between cortisone acetate/hydrocortisone and dual-release hydrocortisone (n = 213), the year of switching was recorded. If a patient received more than one glucocorticoid type or dose during a given year, the type and dose used most frequently that year were recorded. If the patient experienced an adrenal crisis during a given year, the glucocorticoid type and dose in use at the time of the event were recorded. If a patient experienced more than one adrenal crisis in a given year, the type and dose of glucocorticoid used during most events were recorded.

**Figure S1. Serum sodium and potassium in adrenal crisis.** Scatter plot of serum sodium and potassium levels at admission in 1,568 cases with overt and incipient adrenal crisis. Red points represent hypotensive cases, and blue points represent non‑hypotensive cases. To enhance visual clarity, red points were horizontally nudged by +0.5 mmol per liter. A total of 186 cases were excluded due to missing values for one or more variables. Graph was created in GraphPad Prism 10.4.1

**Figure S2. Diagnostic criteria of overt adrenal crisis.** Venn diagram showing diagnostic criteria overlap among 611 cases of overt adrenal crisis with complete data. An additional 80 cases were excluded due to incomplete data and are not shown. Hypotension was present in 62% of cases, while 35% had hyponatremia or hyperkalemia, either isolated or in combination with hypotension. Ten percent of cases met all three diagnostic criteria. Not shown in the diagram are 15 cases (2%) with isolated hypoglycemia and 6 cases (1%) that did not meet the diagnostic criteria but were classified as overt adrenal crisis due to a serious presentation with loss of consciousness. Venn diagram created using R package VennDiagram.

**Figure S3. Incidence of adrenal crisis by sex.** Age-adjusted differences between females and males in risks of A) total crises, B) overt crises, and C) incipient crises. Left-hand plots show differences in proportions; right-hand plots show differences in mean number of crises. The plots include pointwise 95% confidence intervals and the line of no difference.

There were indications that age-adjusted sex differences in risks of crises changed over the study period (p-values for interaction with respect to proportions: 0.28, 0.042, and 0.037; with respect to means: 0.24, 0.040, and 0.031, for total, overt, and incipient crises, respectively), with different change patterns for overt and incipient crises. For overt crises, females had a higher risk compared with males during 2010-2015, with no statistically significant differences before or after. For incipient crises, females had an increased risk starting in 2018-19 and throughout to the end of the study period.

**Figure S4. Glycemic control in type 1 diabetes over time.** HbA1c values in patients with autoimmune/idiopathic PAI and type 1 diabetes, n = 151 (shown in red) compared with patients with type 1 diabetes from the Norwegian Diabetes Registry (shown in blue) between 2009 and 2023. Graph created in GraphPad Prism Version 10.4.1.

**Figure S5. Crisis risk by glucocorticoid type (cortisone/hydrocortisone vs. dual‑release hydrocortisone).** Age-adjusted differences between those using dual-release hydrocortisone (DR-HC) and those using cortisone acetate/hydrocortisone (CA/HC) in risks of A) total crises, B) overt crises, and C) incipient crises. Left-hand plots show differences in proportions; right-hand plots show differences in mean number of crises. The plots include pointwise 95% confidence intervals and the line of no difference.

There were indications that age-adjusted differences between those on dual DR-HC and those on CA/HC in the risks of crises changed over time (p-values for interaction with respect to proportions: 0.078, 0.037, and 0.16; with respect to means: 0.034, 0.057, and 0.15, for any, overt, and incipient crises, respectively). The increased risks observed for DR-HC users early in the period when DR-HC became available were diminished at the end of the study period, with the difference in overt‑crisis probability even reversing direction.

**Figure S6. Crisis risk according to hydrocortisone dose.** Incidence rates across the observation period of overt adrenal crisis (A) and incipient adrenal crisis (B) according to dose of hydrocortisone. Dosages of 5-20 mg are shown in blue, 21-30 mg are shown in red and >30 mg in green. Data are shown as mean number of events per 100 person-years. Children younger than 16 years were excluded from this analysis.

**Figure S7. Effect of Summary Care Record status on crisis risk.** Age-adjusted differences between those having PAI registered as critical information in summary care record and those who did not in risks of A) total crises, B) overt crises, and C) incipient crises. Left-hand plots show differences in proportions; right-hand plots show differences in mean number of crises. The plots include pointwise 95% confidence intervals and the line of no difference.

There were indications that age-adjusted effects of having PAI registered in the summary care record on the risks of particularly incipient crises changed over time (p-values for interaction with respect to proportions: 0.13, 0.90, and 0.079; with respect to means: 0.012, 0.85, and 0.008, for any, overt, and incipient crises, respectively). The increased risks for incipient crises (and therefore also any crises) observed among individuals with summary records early in the period after this routine was introduced

diminished over time, then rose again toward the end of the study period.

**Figure S8. Predicted Probability of Access to Injectable Hydrocortisone.**

Access to injectable hydrocortisone among Norwegian patients with adrenal insufficiency, based on prescription retrievals from the Norwegian Prescription Database (2004 onwards) and shelf life (4 years for 100mg, 5 years for 250mg). Because these preparations have limited shelf life and may be replaced or discarded without use, true individual-level availability cannot be directly observed; this measure captures potential access rather than confirmed possession at the time of emergency.

The dataset consisted of 1,040 patients; 21 were excluded for hydrocortisone pump use, 2 for missing prescription data, and 18 for no observations within the analysis window. The remaining 999 patients (792 with prescription retrievals, 207 without) contributed one observation per year during follow-up, yielding 12,044 person-years of observation (2007-2023). For patients diagnosed before 2004, analyses were restricted to 2007 onward to reduce left-censoring bias from unobserved pre-2004 retrievals.

A) Odds ratios with 95% confidence intervals from generalized estimating equations (GEE) model with AR(1) correlation, including calendar year, sex, age, years since diagnosis, and sex×age interaction. Prior crisis history was not a significant predictor (p=0.20) and was not included in the final model.

B) Population-averaged predicted probability by calendar year, marginalized over distributions of sex, age, and years since diagnosis.

C) Sex-stratified (male in blue and female in red with 95% confidence intervals) population-averaged probability by age, marginalized over calendar year and years since diagnosis, with age modeled using natural cubic splines (df = 3).

Linearity assessment showed calendar year could be modeled linearly despite evidence of non-linearity (retained for parsimony), while age required spline modeling due to non-linear effects. Access increased from 34.4% (95% CI: 31.1–37.7%) in 2007 to 67.3% (95% CI: 64.7–70.0%) in 2023, representing a 33.0 percentage point increase (95% CI: 28.7–37.2; p<0.001). Age effects were non-linear with highest access until middle age and rapidly declining access in patients over 60 years, with steeper decline in males than females. Analyses were performed in R v4.3.3 using nnet and visualized with ggplot2.

**Figure S9. Prehospital stress dosing.** Timely prehospital glucocorticoid stress dosing, either oral or injectable, is a key intervention for preventing progression from impending to severe adrenal crisis. We therefore examined temporal trends in prehospital glucocorticoid treatment during 1,538 hospital admissions (excluding 216 debut crisis admissions) for adrenal crisis from 537 Norwegian patients (2000-2023; mean 2.86 crises per patient, median 2, range 1-35). Patient-weighting (weight=1/n_crises) ensured equal contribution per patient, addressing inverse association between crisis frequency and treatment access (1-2 crises: 56.0% treated vs ≥3 crises: 47.6%, difference 8.4 pp, p=0.012).

Solid lines show patient-weighted predicted probabilities from multinomial logistic regression with any prehospital treatment (at least one treatment) shown in blue, no prehospital treatment (both treatments absent) in red, and unknown status (insufficient information) in gray shaded regions show 95% bootstrap confidence intervals (5,000 patient-level replicates). Model selection favored linear temporal trends over splines.

Probability of any treatment increased from 38.7% (95% CI: 32.3–45.3%) in 2000 to 60.1% (95% CI: 55.8–64.3%) in 2023 (absolute change: 21.4 pp, 95% CI: 12.0–30.6; p<0.001). Correspondingly, no prehospital treatment decreased from 43.1% to 27.5% (absolute change: −15.7 pp, 95% CI: −24.9 to −6.4; p=0.002), while unknown status showed a non-significant decrease from 18.2% to 12.4% (−5.7 pp, 95% CI: −12.9 to 1.1, p=0.11). Analyses were performed in R v4.3.3 using nnet and visualized with ggplot2.

**Table S1. Summary of regression analyses**

|  |  | **Number of crises** | | | **At least one crisis** | | |
| --- | --- | --- | --- | --- | --- | --- | --- |
|  |  | **n/n_obs** | **IRR (95% CI)** | **p-value** | **n/n_obs** | **OR (95% CI)** | **p-value** |
| **Year^1a)^** | Any | 1,012/14,820 |  | <0.001 | 1,012/14,821 |  | <0.001 |
|  | Overt | 1,012/14,821 |  | <0.001 | 1,012/14,821 |  | 0.002 |
|  | Incipient | 1,012/14,820 |  | <0.001 | 1,012/14,820 |  | <0.001 |
|  |  |  |  |  |  |  |  |
| **Year** | Any |  | 1.04 (1.03, 1.05) | <0.001 |  | 1.04 (1.03, 1.05) | <0.001 |
|  | Overt |  | 1.03 (1.01, 1.05) | 0.001 |  | 1.03 (1.01, 1.05) | 0.001 |
|  | Incipient |  | 1.05 (1.04, 1.06) | <0.001 |  | 1.05 (1.04, 1.06) | <0.001 |
|  |  |  |  |  |  |  |  |
| **Year^3)^** | Any |  | 1.04 (1.03, 1.06) | <0.001 |  | 1.04 (1.03, 1.05) | <0.001 |
|  | Overt |  | 1.03 (1.01, 1.05) | 0.001 |  | 1.03 (1.01, 1.05) | 0.002 |
|  | Incipient |  | 1.05 (1.04, 1.07) | <0.001 |  | 1.05 (1.04, 1.07) | <0.001 |
|  |  |  |  |  |  |  |  |
| **Age^1b)^** | Any | 1,012/14,820 |  | <0.001 | 1,012/14,821 |  | <0.001 |
|  | Overt | 1,012/14,821 |  | <0.001 | 1,012/14,821 |  | <0.001 |
|  | Incipient | 1,012/14,820 |  | <0.001 | 1,012/14,820 |  | <0.001 |
|  |  |  |  |  |  |  |  |
| **Age^1b,2)^** | Any |  |  | <0.001 |  |  | <0.001 |
|  | Overt |  |  | <0.001 |  |  | <0.001 |
|  | Incipient |  |  | <0.001 |  |  | <0.001 |
|  |  |  |  |  |  |  |  |
| **Age^1b,4)^** | Any |  |  | 0.32 |  |  | 0.28 |
|  | Overt |  |  | 0.98 |  |  | 0.95 |
|  | Incipient |  |  | 0.090 |  |  | 0.10 |
|  |  |  |  |  |  |  |  |
| **Age groups (years)** |  |  |  |  |  |  |  |
|  | Any |  |  | <0.001 |  |  | <0.001 |
| 2-19 |  |  | 2.03 (1.31, 3.7) | 0.002 |  | 1.86 (1.15, 3.01) | 0.012 |
| 20-39 |  |  | 1.69 (1.40, 2.04) | <0.001 |  | 1.74 (1.43, 2.12) | <0.001 |
| 40-59 |  |  | 1 (ref) |  |  | 1 (ref) |  |
| 60-79 |  |  | 1.32 (1.09, 1.59) | 0.005 |  | 1.25 (1.02, 1.53) | 0.031 |
| 80-99 |  |  | 2.93 (2.14, 4.02) | <0.001 |  | 2.84 (2.01, 4.00) | <0.001 |
|  |  |  |  |  |  |  |  |
|  | Overt |  |  | <0.001 |  |  | <0.001 |
| 2-19 |  |  | 2.22 (1.11, 4.46) | 0.025 |  | 2.55 (1.27, 5.13) | 0.009 |
| 20-39 |  |  | 1.91 (1.42, 2.58) | <0.001 |  | 1.96 (1.44, 2.67) | <0.001 |
| 40-59 |  |  | 1 (ref) |  |  | 1 (ref) |  |
| 60-79 |  |  | 1.39 (1.01, 1.91) | 0.044 |  | 1.33 (0.96, 1.86) | 0.089 |
| 80-99 |  |  | 3.67 (2.29, 5.88) | <0.001 |  | 3.61 (2.21, 5.91) | <0.001 |
|  |  |  |  |  |  |  |  |
|  | Incipient |  |  | <0.001 |  |  | <0.001 |
| 2-19 |  |  | 2.01 (1.22, 3.33) | 0.006 |  | 1.91 (1.12, 3.26) | 0.017 |
| 20-39 |  |  | 1.61 (1.29, 1.99) | <0.001 |  | 1.69 (1.35, 2.11) | <0.001 |
| 40-59 |  |  | 1 (ref) |  |  | 1 (ref) |  |
| 60-79 |  |  | 1.25 (1.01, 1.55) | 0.044 |  | 1.20 (0.95, 1.50) | 0.12 |
| 80-99 |  |  | 2.56 (1.77, 3.69) | <0.001 |  | 2.72 (1.85, 4.01) | <0.001 |
|  |  |  |  |  |  |  |  |
| **Age groups (years)^2)^** |  |  |  |  |  |  |  |
|  | Any |  |  | <0.001 |  |  | <0.001 |
| 2-19 |  |  | 2.46 (1.58, 3.82) | <0.001 |  | 2.23 (1.37, 3.64) | 0.001 |
| 20-39 |  |  | 1.88 (1.56, 2.27) | <0.001 |  | 1.91 (1.56, 2.34) | <0.001 |
| 40-59 |  |  | 1 (ref) |  |  | 1 (ref) |  |
| 60-79 |  |  | 1.13 (0.93, 1.37) | 0.20 |  | 1.09 (0.89, 1.34) | 0.40 |
| 80-99 |  |  | 2.22 (1.62, 3.06) | <0.001 |  | 2.26 (1.59, 3.20) | <0.001 |
|  |  |  |  |  |  |  |  |
|  | Overt |  |  | <0.001 |  |  | <0.001 |
| 2-19 |  |  | 2.51 (1.25, 5.04) | 0.010 |  | 2.88 (1.42, 5.84) | 0.003 |
| 20-39 |  |  | 2.03 (1.50, 2.74) | <0.001 |  | 2.08 (1.52, 2.85) | <0.001 |
| 40-59 |  |  | 1 (ref) |  |  | 1 (ref) |  |
| 60-79 |  |  | 1.32 (0.95, 1.82) | 0.094 |  | 1.26 (0.90, 1.77) | 0.17 |
| 80-99 |  |  | 3.26 (2.02, 5.25) | <0.001 |  | 3.23 (1.96, 5.31) | <0.001 |
|  |  |  |  |  |  |  |  |
|  | Incipient |  |  | <0.001 |  |  | <0.001 |
| 2-19 |  |  | 2.39 (1.45, 3.95) | 0.001 |  | 2.29 (1.34, 3.93) | 0.003 |
| 20-39 |  |  | 1.77 (1.43, 2.20) | <0.001 |  | 1.84 (1.47, 2.31) | <0.001 |
| 40-59 |  |  | 1 (ref) |  |  | 1 (ref) |  |
| 60-79 |  |  | 1.05 (0.85, 1.31) | 0.64 |  | 1.02 (0.81, 1.29) | 0.86 |
| 80-99 |  |  | 1.91 (1.32, 2.76) | 0.001 |  | 2.12 (1.43, 3.13) | <0.001 |
|  |  |  |  |  |  |  |  |
| **DM1** | Any | 1,012/14,820 | 1.58 (1.22, 2.05) | 0.001 | 1,012/14,821 | 1.54 (1.18, 2.03) | 0.002 |
|  | Overt | 1,012/14,821 | 2.03 (1.35, 2.85) | <0.001 | 1,012/14,821 | 1.93 (1.31, 2.83) | 0.001 |
|  | Incipient | 1,012/14,820 | 1.35 (1.01, 1.79) | 0.040 | 1,012/14,820 | 1.47 (1.09, 1.98) | 0.012 |
|  |  |  |  |  |  |  |  |
| **DM1^2)^** | Any |  | 1.46 (1.13, 1.89) | 0.004 |  | 1.45 (1.10, 1.90) | 0.007 |
|  | Overt |  | 1.96 (1.35, 2.85) | <0.001 |  | 1.87 (1.27, 2.74) | 0.001 |
|  | Incipient |  | 1.35 (1.01, 1.79) | 0.040 |  | 1.38 (1.02, 1.85) | 0.034 |
|  |  |  |  |  |  |  |  |
| **DM1^4)^** | Any |  |  | 0.88 |  |  | 0.95 |
|  | Overt |  |  | 0.70 |  |  | 0.58 |
|  | Incipient |  |  | 0.84 |  |  | 0.98 |
|  |  |  |  |  |  |  |  |
| **DM1^2,3)^** | Any |  | 1.51 (1.17, 1.94) | 0.002 |  | 1.49 (1.14, 1.95) | 0.004 |
|  | Overt |  | 2.09 (1.45, 3.01) | <0.001 |  | 1.98 (1.35, 2.90) | <0.001 |
|  | Incipient |  | 1.37 (1.04, 1.82) | 0.028 |  | 1.41 (1.05, 1.89) | 0.024 |
|  |  |  |  |  |  |  |  |
| **DM1^2,5)^** | Any |  |  | 0.55 |  |  | 0.23 |
|  | Overt |  |  | 0.69 |  |  | 0.59 |
|  | Incipient |  |  | 0.72 |  |  | 0.47 |
|  |  |  |  |  |  |  |  |
| **Sex (female)** | Any | 1,012/14,820 | 1.21 (0.98, 1.51) | 0.078 | 1,012/14,821 | 1.18 (0.95, 1.47) | 0.14 |
|  | Overt | 1,012/14,821 | 1.11 (0.81, 1.51) | 0.53 | 1,012/14,821 | 1.10 (0.80, 1.51) | 0.58 |
|  | Incipient | 1,012/14,820 | 1.20 (0.95, 1.51) | 0.14 | 1,012/14,820 | 1.14 (0.90, 1.45) | 0.28 |
|  |  |  |  |  |  |  |  |
| **Sex (female)^2)^** | Any |  | 1.20 (0.97, 1.49) | 0.089 |  | 1.17 (0.94, 1.46) | 0.15 |
|  | Overt |  | 1.10 (0.80, 1.50) | 0.56 |  | 1.09 (0.79, 1.50) | 0.60 |
|  | Incipient |  | 1.18 (0.94, 1.49) | 0.15 |  | 1.13 (0.89, 1.43) | 0.30 |
|  |  |  |  |  |  |  |  |
| **Sex (female)^4)^** | Any |  |  | 0.051 |  |  | 0.11 |
|  | Overt |  |  | 0.045 |  |  | 0.045 |
|  | Incipient |  |  | 0.011 |  |  | 0.019 |
|  |  |  |  |  |  |  |  |
| **Sex (female)^2,3)^** | Any |  | 1.27 (1.03, 1.57) | 0.027 |  | 1.24 (1.00, 1.55) | 0.051 |
|  | Overt |  | 1.13 (0.82, 1.55) | 0.45 |  | 1.13 (0.82, 1.56) | 0.45 |
|  | Incipient |  | 1.26 (1.00, 1.58) | 0.050 |  | 1.21 (0.95, 1.53) | 0.12 |
|  |  |  |  |  |  |  |  |
| **Sex (female)^3,4)^** | Any |  |  | 0.24 |  |  | 0.28 |
|  | Overt |  |  | 0.040 |  |  | 0.042 |
|  | Incipient |  |  | 0.031 |  |  | 0.037 |
|  |  |  |  |  |  |  |  |
| **Dual release hydrocortisone vs. cortisone acetate /hydrocortisone^2^** | Any | 925/7,682 | 1.27 (0.98, 1.64) | 0.066 | 925/7,683 | 1.32 (0.99, 1.75) | 0.055 |
|  | Overt | 925/7,683 | 0.95 (0.60, 1.49) | 0.82 | 925/7,683 | 0.76 (0.46, 1.26) | 0.28 |
|  | Incipient | 925/7,682 | 1.44 (1.09, 1.90) | 0.011 | 925/7,682 | 1.54 (1.15, 2.07) | 0.004 |
|  |  |  |  |  |  |  |  |
| **Dual release hydrocortisone vs. cortisone acetate /hydrocortisone^2)^** | Any |  | 1.21 (0.93, 1.57) | 0.16 |  | 1.27 (0.95, 1.71) | 0.11 |
|  | Overt |  | 0.95 (0.60, 1.52) | 0.85 |  | 0.76 (0.45, 1.29) | 0.31 |
|  | Incipient |  | 1.35 (1.01, 1.80) | 0.042 |  | 1.46 (1.07, 1.98) | 0.017 |
|  |  |  |  |  |  |  |  |
| **Dual release hydrocortisone vs. cortisone acetate /hydrocortisone^4)^** | Any |  |  | 0.017 |  |  | 0.046 |
|  | Overt |  |  | 0.042 |  |  | 0.027 |
|  | Incipient |  |  | 0.11 |  |  | 0.12 |
|  |  |  |  |  |  |  |  |
| **Dual release hydrocortisone vs. cortisone acetate /hydrocortisone^3)^** | Any |  | 1.35 (1.05, 1.73) | 0.021 |  | 1.39 (1.05, 1.84) | 0.022 |
|  | Overt |  | 1.04 (0.66, 1.62) | 0.87 |  | 0.83 (0.50, 1.37) | 0.46 |
|  | Incipient |  | 1.49 (1.13, 1.97) | 0.005 |  | 1.62 (1.20, 2.17) | 0.001 |
|  |  |  |  |  |  |  |  |
| **Dual release hydrocortisone vs. cortisone acetate /hydrocortisone^2,3)^** | Any |  | 1.29 (0.99, 1.68) | 0.057 |  | 1.35 (1.01, 1.81) | 0.046 |
|  | Overt |  | 1.06 (0.67, 1.69) | 0.80 |  | 0.85 (0.50, 1.43) | 0.53 |
|  | Incipient |  | 1.41 (1.06, 1.88) | 0.020 |  | 1.53 (1.13, 2.08) | 0.007 |
|  |  |  |  |  |  |  |  |
| **Dual release hydrocortisone vs. cortisone acetate /hydrocortisone^3,4)^** | Any |  |  | 0.034 |  |  | 0.078 |
|  | Overt |  |  | 0.057 |  |  | 0.037 |
|  | Incipient |  |  | 0.15 |  |  | 0.16 |
|  |  |  |  |  |  |  |  |
| **Dual release hydrocortisone vs. cortisone acetate /hydrocortisone^22,5)^** | Any |  |  | 0.45 |  |  | 0.20 |
|  | Overt |  |  | 0.057 |  |  | 0.028 |
|  | Incipient |  |  | 0.70 |  |  | 0.71 |
|  |  |  |  |  |  |  |  |
| **Dose HC^1a)^** | Any | 524/5,418 |  | 0.12 | 524/5,419 |  | 0.064 |
|  | Overt | 524/5,419 |  | 0.28 | 524/5,419 |  | 0.11 |
|  | Incipient | 524/5,418 |  | 0.34 | 524/5,418 |  | 0.26 |
|  |  |  |  |  |  |  |  |
| **Dose HC^1a,2,3)^** | Any |  |  | 0.10 |  |  | 0.043 |
|  | Overt |  |  | 0.41 |  |  | 0.15 |
|  | Incipient |  |  | 0.19 |  |  | 0.13 |
|  |  |  |  |  |  |  |  |
| **Dose fludrocortisone^1a)^** | Any | 514/5,622 |  | 0.88 | 514/5,623 |  | 0.75 |
|  | Overt | 514/5,623 |  | 0.39 | 514/5,623 |  | 0.39 |
|  | Incipient | 514/5,622 |  | 0.89 | 514/5,622 |  | 0.80 |
|  |  |  |  |  |  |  |  |
| **Dose fludrocortisoene^1a,2,3)^** | Any |  |  | 0.87 |  |  | 0.69 |
|  | Overt |  |  | 0.29 |  |  | 0.30 |
|  | Incipient |  |  | 0.98 |  |  | 0.80 |
|  |  |  |  |  |  |  |  |
| **PAI registered in summary record** | Any | 859/5,240 | 1.09 (0.88, 1.35) | 0.45 | 859/5,240 | 1.03 (0.81, 1.31) | 0.81 |
|  | Overt | 859/5,240 | 0.58 (0.39, 0.86) | 0.006 | 859/5,240 | 0.62 (0.41, 0.95) | 0.030 |
|  | Incipient | 859/5,240 | 1.36 (1.07, 1.73) | 0.012 | 859/5,240 | 1.31 (1.01, 1.69) | 0.042 |
|  |  |  |  |  |  |  |  |
| **PAI registered in summary record^2)^** | Any |  | 1.12 (0.87, 1.43) | 0.38 |  | 1.04 (0.79, 1.36) | 0.78 |
|  | Overt |  | 0.57 (0.37, 0.87) | 0.010 |  | 0.60 (0.38, 0.97) | 0.036 |
|  | Incipient |  | 1.42 (1.08, 1.86) | 0.012 |  | 1.34 (1.00, 1.79) | 0.047 |
|  |  |  |  |  |  |  |  |
| **PAI registered in summary record^4)^** | Any |  |  | 0.013 |  |  | 0.14 |
|  | Overt |  |  | 0.86 |  |  | 0.91 |
|  | Incipient |  |  | 0.008 |  |  | 0.080 |
|  |  |  |  |  |  |  |  |
| **PAI registered in summary record^3)^** | Any |  | 1.09 (0.88, 1.35) | 0.44 |  | 1.03 (0.81, 1.30) | 0.83 |
|  | Overt |  | 0.58 (0.39, 0.85) | 0.006 |  | 0.61 (0.40, 0.94) | 0.024 |
|  | Incipient |  | 1.35 (1.07, 1.72) | 0.013 |  | 1.31 (1.01, 1.69) | 0.042 |
|  |  |  |  |  |  |  |  |
| **PAI registered in summary record^2,3)^** | Any |  | 1.13 (0.89, 1.44) | 0.32 |  | 1.04 (0.80, 1.36) | 0.77 |
|  | Overt |  | 0.57 (0.37, 0.88) | 0.011 |  | 0.60 (0.37, 0.96) | 0.032 |
|  | Incipient |  | 1.42 (1.09, 1.86) | 0.011 |  | 1.35 (1.01, 1.80) | 0.042 |
|  |  |  |  |  |  |  |  |
| **PAI registered in summary record^3,4)^** | Any |  |  | 0.012 |  |  | 0.13 |
|  | Overt |  |  | 0.85 |  |  | 0.90 |
|  | Incipient |  |  | 0.008 |  |  | 0.079 |
|  |  |  |  |  |  |  |  |

Results from multilevel negative binomial and binary logistic regression analyses, including patient specific random intercepts. All p-values are from Wald tests. The numbers of included patients/observations are indicated for each analysis. The results presented on dark background are also reported in Main Table 2 and repeated here for completeness. Analyses of dual-release hydrocortisone versus cortisone acetate or hydrocortisone, and of Summary Care Record data, include observations from 2013 and 2017 onward, respectively. Analyses of hydrocortisone and fludrocortisone doses include patients aged ≥ 16 years.

^1a)^ Modeled non-linearly using restricted cubic splines with three knots

^1b)^ Modeled non-linearly using restricted cubic splines with four knots

^2)^ Adjusted for year (non-linear)

^3)^ Adjusted for age (non-linear)

^4)^ Tested for interactions with year (non-linear)

^5)^ Tested for interactions with age (non-linear)

Abbreviations: n, number of patients; n obs, number of observations; IRR, incidence rate ratio; CI, confidence interval; OR, odds ratio; y, years of age; DM1, diabetes mellitus type 1; HC, hydrocortisone. Medication type compares dual release hydrocortisone with cortisone acetate/hydrocortisone.
